# Imbalance in Coagulation/Fibrinolysis Inhibitors Resulting in Extravascular Thrombin Generation in Gliomas of Varying Levels of Malignancy

**DOI:** 10.3390/biom11050663

**Published:** 2021-04-29

**Authors:** Marek Z. Wojtukiewicz, Marta Mysliwiec, Elwira Matuszewska, Stanislaw Sulkowski, Lech Zimnoch, Barbara Politynska, Anna M. Wojtukiewicz, Stephanie C. Tucker, Kenneth V. Honn

**Affiliations:** 1 Department of Oncology, Medical University of Białystok, 12 Ogrodowa St., 15-027 Bialystok, Poland; marta.mysl@gmail.com; 2 Department of Clinical Oncology, Comprehensive Cancer Center, 12 OgrodowaSt., 15-369 Bialystok, Poland; emat22@wp.pl; 3 Department of General Pathomorphology, Medical University of Bialystok, Waszyngtona 13, 15-269 Bialystok, Poland; sulek2854@gmail.com; 4 Department of Medical Pathomorphology, Medical University of Bialystok, 15-269 Bialystok, Poland; lzimnoch@adres.pl; 5 Department of Philosophy and Human Psychology, Medical University of Bialystok, 15-295 Bialystok, Poland; bpolitynska@wp.pl (B.P.); aniawojtukiewicz@gmail.com (A.M.W.); 6 Robinson College, University of Cambridge, Cambridge CB3 9AN, UK; 7 Bioactive Lipids Research Program, Department of Pathology-School of Medicine, Wayne State University, Detroit, MI 48202, USA; stucker@med.wayne.edu; 8 Karmanos Cancer Institute, Detroit, MI 48201, USA; 9 Department of Chemistry, Wayne State University, Detroit, MI 48202, USA; k.v.honn@wayne.edu; 10 Department of Oncology, Wayne State University, Detroit, MI 48202, USA

**Keywords:** blood coagulation/fibrinolysis inhibitors, hemostasis, F1+2, TFPI, TFPI-2, PC, PS, thrombomodulin, PAI-1, gliomas, glial tumors

## Abstract

Neoplastic processes are integrally related to disturbances in the mechanisms regulating hemostatic processes. Brain tumors, including gliomas, are neoplasms associated with a significantly increased risk of thromboembolic complications, affecting 20–30% of patients. As gliomas proliferate, they cause damage to the brain tissue and vascular structures, which leads to the release of procoagulant factors into the systemic circulation, and hence systemic activation of the blood coagulation system. Hypercoagulability in cancer patients may be, at least in part, a result of the inadequate activity of coagulation inhibitors. The aim of the study was to evaluate the expression of the inhibitors of the coagulation and fibrinolysis systems (tissue factor pathway inhibitor, TFPI; tissue factor pathway inhibitor-2 TFPI-2; protein C, PC; protein S, PS, thrombomodulin, TM; plasminogen activators inhibitor, PAI-1) in gliomas of varying degrees of malignancy. Immunohistochemical studies were performed on 40 gliomas, namely on 13 lower-grade (G2) gliomas (8 astrocytomas, 5 oligodendrogliomas) and 27 high-grade gliomas (G3–12 anaplastic astrocytomas, 4 anaplastic oligodendrogliomas; G4–11 glioblastomas). A strong expression of TFPI-2, PS, TM, PAI-1 was observed in lower-grade gliomas, while an intensive color immunohistochemical (IHC) reaction for the presence of TFPI antigens was detected in higher-grade gliomas. The presence of PC antigens was found in all gliomas. Prothrombin fragment 1+2 was observed in lower- and higher-grade gliomas reflecting local activation of blood coagulation. Differences in the expression of coagulation/fibrinolysis inhibitors in the tissues of gliomas with varying degrees of malignancy may be indicative of their altered role in gliomas, going beyond that of their functions in the hemostatic system.

## 1. Introduction

Neoplastic processes are integrally related to disturbances in the mechanisms regulating hemostatic processes and thromboembolism may be the earliest clinical symptom of cancer. Brain tumors, including gliomas, are neoplasms associated with a significantly increased risk of thromboembolic complications, affecting 20–30% of patients [1,2]. Numerous studies have shown that glioma tissues are a rich source of tissue factor (TF) [3,4]. TF is the key procoagulant in malignancy [4,5,6]. The proliferation and invasion of gliomas, causes damage to brain tissue and vascular structures and lead to the release of procoagulant factors into the systemic circulation, and thereby systemic activation of the coagulation system [7]. Furthermore, clinical management of brain tumors, in the form of surgery, radiotherapy, and chemotherapy, results in damage to brain tissue and endothelial cells, thus increasing the production and release of TF. Fibrin is thought to be deposited within and around the foci of glioblastomas (GBM), suggesting that the TF present on tumor cells may be enzymatically active [8]. 

Deficient activity of coagulation inhibitors may, at least partially, be responsible for hypercoagulability in cancer patients. Tissue factor pathway inhibitor (TFPI), which inhibits the activity of both coagulation factor Xa and tissue factor/factor VIIa (TF/VIIa) complex, is one of the most important inhibitors of blood coagulation [9]. Plasma concentrations of TFPI have been shown to be increased in patients suffering from GBM or different cancers (especially at the terminal stage of the disease) in comparison to healthy individuals [10,11,12]. It is thus reasonable to suspect that high plasma TFPI concentration may indicate a host compensatory mechanism resulting from a hypercoagulable state in cancer patients. Indeed, it has been suggested that tumor-associated TFPI may play a role in the reduction of hematogenous metastasis [13]. Anticancer treatment (surgery, radiotherapy, or chemotherapy) commonly leads to the normalization of TFPI plasma levels [14,15].

Whilst similar in structure to its homologue TFPI, TFPI-2 displays a distinctly different inhibitory spectrum from that of the former. Increasing degrees of tumor malignancy is associated with the expression of TFPI-2, possibly suggesting a role for TFPI-2 in maintaining the stability of the tumor environment and inhibiting the growth of neoplasms and the formation of metastases. Correspondingly, TFPI-2 mRNA has been shown to be absent in choriocarcinoma [16], fibrosarcoma [17], and pancreatic cancer [18] cell lines. TFPI-2 has also been demonstrated to stimulate apoptosis in GBM cancer cells [19] and to inhibit angiogenesis in experimental models [20,21].

An important inhibitory system limiting the activation of coagulation is the protein C system, which includes protein C (PC), protein S (PS), thrombomodulin (TM), and endothelial cell protein C receptor (ECPCR). Active PC, together with the participation of PS, has an anticoagulant effect by inactivating factors Va and VIIIa and increasing the fibrinolytic activity of endothelial cells by inhibiting PAI-1 [22]. PC activation depends on the formation of a complex of thrombin and TM. When attached to TM, activated protein C (APC) is formed [23]. PC activation can also take place in the absence of TM, through binding to ECPCR [22]. TM expression declines with advancing clinical stages of the disease and tumor size, and with decreasing tumor cell differentiation [24]. It is of interest that, as with TFPI-2, silencing of the TM gene promoter is implicated in the observed downregulation of TM synthesis [24]. It has been documented that this protein regulates cancer growth independently of its anticoagulant activity [25]. In turn, PS is produced and released by the smooth muscle cells of blood vessels and has mitogenic activity in relation to these cells [26,27]. This suggests that apart from anticoagulant activity (through interaction with protein C), protein S may participate in the repair of blood vessels via an autocrine mechanism. Similar to PC, PS exerts antiapoptotic activity toward hypoxic neurons as well [28]. 

A known regulator of proteolysis is plasminogen activators inhibitor (PAI-1). PAI-1 expression in cancer tissue is related to poor clinical prognosis [29,30,31,32]. PAI-1 protein, which is released in an active form by vascular endothelial cells (amongst others), binds and inactivates urokinase plasminogen activator (u-PA) and tissue plasminogen activator (t-PA), which convert plasminogen to plasmin [33]. PAI-1 also exerts its activity through a mechanism that is independent of u-PA/t-PA inhibition. It regulates tumor growth through angiogenesis and is involved in the migration, invasion and adhesion of cancer cells [34,35,36]. In certain forms of cancer (breast and head and neck cancer) multi-drug resistance is associated with high expression of PAI-1 [29,37]. Indeed, studies have indicated that large amounts of PAI-1 are found in GBM, and this protein is related to tumor metastasis [38].

The main enzyme involved in blood coagulation is thrombin, which additionally exhibits multiple relevant biological effects, including an increase in tumor cell adhesiveness and metastatic potential, and induction of tumor cell-induced platelet aggregation (TCIPA) [39,40]. A byproduct in the reaction of thrombin formation is prothrombin fragment F1+2 (F1+2), the presence of which has been shown to be an indicator of local activation of blood coagulation (and thrombin generation) in various tumor types [40,41]. Prothrombin fragment 1+2 (F1+2) is considered to be useful for diagnosis of thrombosis [42].

The aim of the present study was to evaluate the expression of inhibitors of the coagulation and fibrinolytic systems in gliomas of various degrees of malignancy.

## 2. Materials and Methods

Glioma tissues and tissues from the margin of these tumors were obtained at the surgical resection of 40 cancer patients. The material consisted of 13 lower-grade and 27 higher-grade malignant tumors.

Immunohistochemical (IHC) studies were performed on G2-grade gliomas (8 astrocytomas, 5 oligodendrogliomas) and high-grade gliomas (G3–12 anaplastic astrocytomas, 4 anaplastic oligodendrogliomas; G4–11 glioblastomas), as well as control fragments of respective normal tissues, which were derived from the neoplasm-free surgical margins. 

The study protocol was approved by the local Ethics Committee of the Medical University in Bialystok, Poland (approval number R-I-002/256/2003). Informed consent was obtained from the patients.

Antigens were detected with avidin-biotin complex technique (ABC) using reagents (Vectastain Kits, Vector Laboratories, Burlingame, CA, USA), which have been described previously [43]. Polyclonal antibodies against TFPI, TFPI-2, F1+2, PC, PS were produced in rabbits and were kindly provided by Dr. Walter Kisiel (University of New Mexico, Dept. of Pathology, School of Medicine, Albuquerque, NM, USA). The antibodies were employed in our earlier studies [12,41,44,45]. In turn, monoclonal antibody to PAI-1 and polyclonal goat antibody directed to human TM was purchased from American Diagnostica, Greenwich, USA.

The results of staining of the glioma tissues were compared with matched normal tissues, which were processed simultaneously. Antigens of the proteins tested were detected as the brown reaction product of the avidin-biotin complex with the substrate. Visual assessment of protein expression was performed in 10 random high-power fields by two independent observers. 

**Figure 1:**
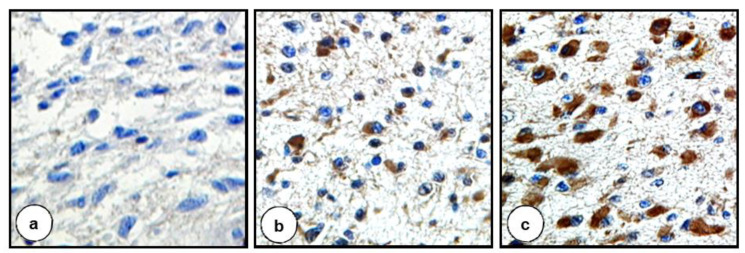
Distinct intensity of staining reflected by different IHC scores. (**a**) IHC score 0 = 100% cells staining negatively. (**b**) IHC score 130 = 50% cells staining negatively + 20% cells staining moderately + 30% cells staining strongly. (**c**) IHC score 270 = 10% cells staining negatively + 90% cells staining strongly. Original magnification ×400 (**a**,**c**), ×200 (**b**).

The intensity of IHC reactions was evaluated according to Hirsch et al. [46] with the modification by Pirker et al. [47,48,49]. A score for each tissue core was generated using a semi-quantitative approach according to the following algorithm: the percentage of positive tumor cells per slide (0–100%) was multiplied by the dominant intensity pattern of staining (0—negative for trace; 1—weak; 2—moderate; 3—intense). Hence the range for the overall score was 0–300. Specimens with a score of 0–199 were classified as being negative, while those with a score between 200–300 as positive. The IHC score was calculated based on the following formula: 1x (percentage of cells staining weakly (1+)) +2 × (percentage of cells staining moderately (2+) + 3 × (percentage of cells staining strongly (3+)) [47,48,49]. Examples of different intensities of IHC staining are depicted in Figure 1. 

The χ^2^ test was employed for statistical analysis. A *p* value of <0.05 was considered statistically significant.

## 3. Results

**Figure 2:**
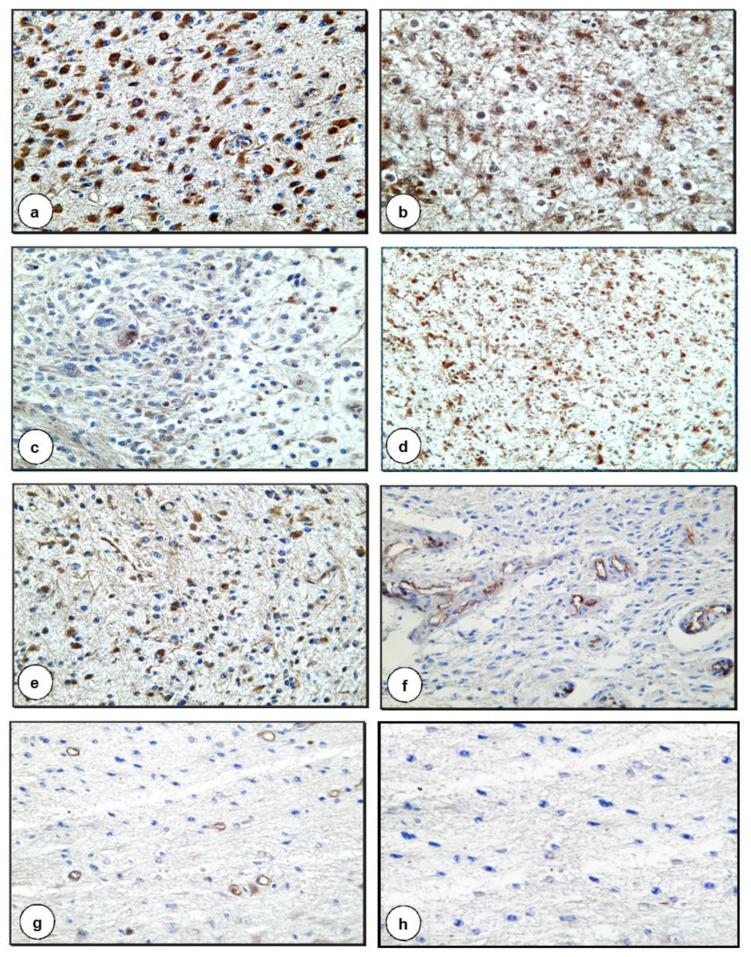
Expression of coagulation and fibrinolysis inhibitors in G2 and G3 astrocytomas (**a**) Positive IHC reaction for the presence of PC in cancer cells in G3 astrocytoma (×400) (**b**) Positive IHC staining for the presence of TM in neoplastic cells in G3 astrocytoma (×400) (**c**) Positive IHC reaction for the presence of PAI-1 in cancer cells in G3 astrocytoma (×200) (**d**) Positive IHC staining for the presence of TFPI-2 in neoplastic cells in G2 astrocytoma (×100) (**e**) Positive IHC reaction for the presence of F1+2 in cancer cells in G3 astrocytoma (×200) (**f**) Positive IHC staining for the presence of TFPI in endothelial cells in G2 astrocytoma (×200) (**g**) Positive IHC reaction for the presence of PS in endothelial cells in G2 astrocytoma (×100) (**h**) Negative control (×200).

The results of the expression of coagulation/fibrinolysis inhibitors are presented in Figure 2 and Table 1.

### 3.1. TFPI

The extent of the expression of TFPI connected with tumor cells was proportional to the grade of malignancy, i.e., the strongest expression was characteristic of glioma cells of the highest-grade malignancy. In contrast, expression of TFPI antigens associated with vascular endothelial cells was pronounced in glial cells of lower-grade malignancy (Figure 2f, Table 1) and in areas of vascular hyperplasia in gliomas of higher-grade malignancy. 

### 3.2. TFPI-2

A different pattern was identified in the case of the TFPI-2 antigen, which had the strongest expression in low-grade gliomas. In these tumors, a strong positive association was observed with neoplastic cells (Figure 2d, Table 1) and a weaker, though nonetheless pronounced, positive reaction in the neuropil. A similar expression of TFPI-2 antigens was observed in the tissues of G2 and G3 oligodendrogliomas. In addition, TFPI-2 antigens were identified in the vascular walls of gliomas of various levels of malignancy. Expression of the antigen was associated with endothelial cells and pericytes. A strong color reaction was observed, above all, in areas of vascular hyperplasia. 

### 3.3. PC

The presence of PC antigens was found in the cytoplasm of neoplastic cells of all gliomas (Figure 2a, Table 1), and the intensity of the color reaction was proportional to the degree of pleomorphism of the neoplastic cells. PC was also clearly expressed in association with vascular endothelial cells in gliomas of higher-grade malignancy and there were traces in the small blood vessels of lower-grade gliomas. 

### 3.4. PS

PS antigens were found to be associated with tumor cells and in the walls of small blood vessels of gliomas of lower-grade malignancy (Figure 2g, Table 1). 

### 3.5. TM

The presence of TM antigens was found in neoplastic glioma cells of various degrees of malignancy (Figure 2b, Table 1), though the strongest expression was seen in lower-grade astrocytoma neoplastic cells and oligodendroglioma cells.

### 3.6. PAI-1

PAI-1 antigens were clearly present in neoplastic cells, in the endothelial cells of small blood vessels and in the neuropil of gliomas of lower-grade malignancy and G2 and G3 oligodendrogliomas. In cancer cells in G3 astrocytomas, the reaction was less intense (Figure 2c, Table 1).

### 3.7. F1+2

The presence of thrombin was assessed indirectly by attempting to identify antigens of the F1+2 fragment of prothrombin. A clear positive reaction for F1+2 was observed in astrocytomas of higher-grade malignancy in the cytoplasm of neoplastic cells (Figure 2e, Table 1) and the vascular wall. A weaker reaction was seen in the neoplastic extensions of stellate glial cells. This reaction, however, was significantly stronger than in the normal tissue surrounding the tumor. In G3 oligodendrogliomas, the reaction was less intense, it occurred only in some neoplastic cells, and it resembled that observed in low-grade gliomas.

## 4. Discussion

There exists a complex relationship between cancer cells and components of the hemostatic system. In fact, cancer patients demonstrate an increased tendency for hypercoagulation, which in itself is compounded by chemotherapy and surgical interventions. Blood coagulation may be directly activated by cancer cells which are able to interact with platelets and components of the blood coagulation system, generating thrombin, which is highly instrumental in stimulating tumor growth and metastatic dissemination [50]. Activation of clotting may be seen as a special type of inflammatory response to a variety of stimuli, such as sustaining damage to a vessel wall, intravascular cell aggregation, or entry of abnormal cells such as tumor cells into the blood. The balance between the coagulation and fibrinolytic systems can readily change, through an excess of tissue factor (TF), to a prothrombotic state in cancer, and lead to fibrin formation and deposition at the site of the malignancy. Thromboembolic complications in cancer patients may result from profiles of oncogenic driver mutations and their impact on the expression of coagulation-related genes—coagulome [51,52]. A precarious balance between blood coagulation and fibrinolysis is regulated by coagulation/fibrinolysis inhibitors [53].

In the present study, thrombin generation was assessed indirectly by looking for antigens of the F1+2 fragment of prothrombin. The F1+2 fragment is a byproduct of the conversion of prothrombin to thrombin and is an indicator of extravascular thrombin generation in neoplasms [41]. The clear presence of the F1+2 antigen in the vascular wall, neuropil, and higher-grade gliomas in connection with tumor cells suggests the formation of thrombin in loco and may indicate that this process contributes to the progression of gliomas. The results reported above do indeed suggest that clotting is activated in loco. The presence of F1+2 in association with colon cancer cells and the endothelial cells of blood vessels supplying the tumor indicates that thrombin is generated at the sites [54]. In turn, the high plasma concentrations of F1+2 reflect a high risk for thrombosis [42,55].

An attempt was made to evaluate the expression of proteins involved in anticoagulant mechanisms. The role of PC system factors in gliomas has not yet been established. In the authors’ own research, variable expression of the factors of the PC system in gliomas was found, depending on the degree of malignancy. The expression of PC antigens in higher-grade gliomas and the expression of PS and TM antigens in lower-grade gliomas observed in vascular endothelial cells and neoplastic cells may indicate impairment of endothelial anticoagulant function in both higher and lower-grade gliomas. The established expression of TM in normal brain tissue, both on the surface of astrocytes and in small blood vessels, may indicate the activation of PC in a mechanism dependent on the formation of a complex of thrombin with TM, and thus the role of TM in the regulation of cerebral microcirculation hemostasis under physiological conditions [56]. 

TM expression diminishes with increasing clinical stage of the disease and tumor size, as well as decreasing cancer cell differentiation [24]. In our study, stronger TM expression was associated with lower-grade malignant glioma tissues. It is possible that TM expression, along with an increase in tumor malignancy, can be compensated for by the presence of the second receptor determining PC activation—EPCR [22]. Tsuneyoshi et al. [57] confirmed the expression of EPCR in studies on human GBM cell lines, while no EPCR antigens were found on cell lines of other brain tumors. In low-grade gliomas, PC can be activated with TM, and in higher-grade gliomas with EPCR. It has been shown that activation of PC in an EPCR-dependent mechanism leads to inhibition of TF expression on U937 leukemia cell lines [58]. The results of experimental studies suggest that APC forms a complex with PAI-1, which stimulates u-PA and results in the activation of extracellular matrix proteases, thereby leading to a surge in the invasion of tumor cells [59]. Activation of EPCR plays a role in APC-induced increased vascular endothelial cell proliferation and angiogenesis, which are mediated by MAPK (mitogen-activated protein kinase), PI3K (phosphatidylinositol 3-kinase), and eNOS (endothelial nitric oxide synthase) pathways [59]. 

Additionally, the different location of PS in gliomas of different degrees of malignancy may indicate the participation of this factor in the process of their growth. Studies in cell lines have shown that primary brain tumor cells [60] and lung cancer cells [61] synthesize and release active S protein. By contrast, in melanoma [62], prostate cancer [63], gastric cancer [64], and pancreatic cancer [65], no or only subtle PS expression has been observed. In addition, it has been shown that most lung cancer cells expressing PS simultaneously express the Tyro-3 receptor [66]. Tyro-3 belongs to the protein receptors with tyrosine kinase activity, and PS is the ligand of this receptor [67]. The coexistence of PS and its receptor in lung cancer cells may support a hypothesis for the role of PS in the process of tumor growth. Tyro-3 receptor expression has also been demonstrated in normal brain tissue [68]. Tumor-secreted protein S (ProS1) activates a Tyro3-Erk signaling axis and protects cancer cells from apoptosis, and thus supports cancer cell survival [67]. The above studies suggest that PS not only plays a role in the anticoagulation process in gliomas, but is also involved in the tumor growth process, which may constitute the subject of another promising area of research in the future.

In terms of these considerations, the role of the tissue factor-dependent blood coagulation pathway TFPI and TFPI-2 inhibitors in the control of glial tumor growth and in the coagulation process in loco is interesting. Along with the increase in the pleomorphism of neoplastic cells and the degree of histopathological malignancy of the tumor, an increase in TFPI expression and loss of TFPI-2 expression has been found, which indicates a different role for both of the TF inhibitors in the biology of gliomas of varying degrees of malignancy. Most cancers, including non-small cell lung cancer, renal, breast, and colon cancer, have not been shown to express TFPI in association with tumor cells [69]. In contrast, TFPI mRNA and protein have been observed in colon, breast, and pancreatic cancer cell lines [70]. Similarly, in vitro experiments, IHC, and in situ hybridization studies have revealed the presence of TFPI antigen in cancer cells in most cases of colon cancer and in all cases of breast cancer, which may suggest a role for TFPI in the biology of the neoplasms [12]. Moreover, the presence of TFPI has been found in malignant melanoma cells [71]. It has been demonstrated that TFPI regulates the procoagulant function of TF in highly aggressive melanoma and this activity is essential for the perfusion of vasculogenic mimicry channels formed by TF-expressing melanoma cells [72]. In the present study, we report elevated expression of TFPI in higher-grade gliomas. In earlier studies, we also observed high TF expression in higher-grade gliomas [73]. This may suggest a modeling effect of TFPI on TF-dependent signaling functions and thus on tumor growth and progression. In turn, the high expression of TFPI in vascular endothelial cells of gliomas, depending on the degree of malignancy, may indicate the role of this protein in maintaining hemostasis. This is confirmed in a study by Takeshima et al. [74], in which a significantly higher expression of TFPI in tumors with intracranial bleeding was observed compared to tumors without intracranial bleeding. Of interest is the fact that, in an experimental model, Amirkhoshravi et al. [75] observed that administration of TFPI to mouse tissue in the form of IV injection, preceding inoculation of the animals with tumor cells, resulted in reduced tumor cell-induced blood coagulation activation as well as diminished blood-borne lung metastasis.

With regard to TFPI-2, our own results are consistent with the reports of other authors who observed the loss of TFPI-2 expression in glioma tissues with increasing tumor malignancy [76,77,78]. There are reports of TFPI-2 mRNA absence in cancer cell lines of choriocarcinoma, fibrosarcoma, and pancreatic adenocarcinoma [79,80]. In some highly aggressive cancers, deletion of the TFPI-2 gene *locus* on chromosome 7q results in the complete lack of TFPI-2 protein expression. TFPI-2 positive GBM, as well as low-grade glioma cell lines, demonstrated enhanced apoptosis, while in normal glial tissue and in TFPI-2 negative glioma cell lines, apoptosis was absent [17,81]. Interestingly, proapoptotic signaling pathways and apoptosis were observed in human glioma cell lines upon TFPI-2 restoration [19].

In our own study, different expression and localization of PAI-1 antigens in the tissues of gliomas was demonstrated, along with simultaneous PAI-1 deficiency in more malignant gliomas, which may indicate a lack of inhibition of fibrinolysis. This is consistent with our earlier observations regarding the increased expression of D-dimers in cancer cells and tumor stroma in the vicinity of blood vessels in gliomas [73]. Activation of fibrinolysis is a response to increased extravascular coagulation. Studies determining the distribution of PAI-1 in glioma tissues are not unequivocal. In a study of 24 human gliomas of various degrees of malignancy, PAI-1 expression was associated with high-grade glioma neoplastic cells, but no expression of PAI-1 was associated with vascular endothelial cells or with lower-grade glioma neoplastic cells was found [82]. Other studies have shown a strong expression of PAI-1 at the sites of vascular hyperplasia of higher-grade gliomas, indicating the involvement of this protein in the angiogenesis process [82,83]. PAI-1 is overexpressed in glioma tissues and inhibits glioma cell proliferation, invasion, and metastasis through the PAI-1/PI3K/AKT pathway [32]. Clinical observations show a correlation between high levels of PAI-1 and cancer relapse and survival time in patients with gliomas [84]. It has been shown that in glioma tissues, PAI-1 expression increases with their level of malignancy [32,85]. It has been suggested that targeting PAI-1 may constitute an important strategy for the treatment of GBM [38].

The results of our own research suggest that local activation of coagulation takes place in glioma tissues, and that inhibitors of the hemostatic system are not able to ensure the appropriate but precarious balance between blood coagulation and fibrinolysis. The different expression of coagulation/fibrinolysis inhibitors in the tissues of gliomas with different degrees of malignancy may indicate their distinct role in gliomas, going beyond their functions in the hemostatic system. In gliomas of higher-grade malignancy, there is a multidirectional failure of the anticoagulant mechanisms expressed by dysregulation in the PC system and PAI-1 deficiency. TF-dependent coagulation pathway inhibitors seem to play a different role in the biology of gliomas. Namely, TFPI is involved in inhibiting the pro-coagulatory activity of TF, but it does not balance the activity of TF, while TFPI-2 seems to be a factor regulating the processes of tumor growth and progression. In summary, our preliminary analysis of blood coagulation/fibrinolysis inhibitors in gliomas of different malignancy may point to their role in the growth and progression of glial tumors.

The results of the study warrant further and more detailed research employing quantitative methods, such as Western blotting or real-time PCR. Moreover, numerous hemostatic system proteins are phosphorylated or exhibit other post-translational modifications, and it would be advantageous for additional studies to capitalize on this, using Western blotting or IHC with more specific antibodies (e.g., phosphospecific antibodies). Studies of this kind could contribute to the development of novel therapies for gliomas, replacing conventional anticoagulation in the treatment or prevention of venous thromboembolic events.

## Figures and Tables

**Table 1:** Number of tumors exhibiting distinct intensity of IHC reactions towards blood coagulation/fibrinolysis inhibitors in gliomas of different malignancy.

Coagulation Factors	Localization	Low-Grade Gliomas (n = 13) IHC Score		High-Grade Gliomas (n = 27) IHC Score		*p* Value
		<200	≥200	<200	≥200	
**F1+2**	**Cancer cells**	9	4	2	25	<0.001
	Tumor stroma in the vicinity of blood vessels	4	9	8	19	NS
**TFPI**	Cancer cells	9	4	7	20	<0.01
	Endothelial cells	1	12	1	26	NS
**TFPI-2**	Cancer cells	0	13	25	2	<0.001
	Endothelial cells	3	10	13	14	NS
**PAI-1**	Cancer cells	1	12	24	3	<0.001
	Endothelial cells	1	12	23	4	<0.001
**PC**	Cancer cells	5	8	0	27	<0.001
	Endothelial cells	5	8	0	27	<0.001
**PS**	Cancer cells	2	11	23	4	<0.001
	Endothelial cells	2	11	2	25	NS
**TM**	Cancer cells	1	12	8	19	NS
	Endothelial cells	4	9	26	1	<0.001

NS—not significant.

## Data Availability

Data supporting reported results can be obtained from the corresponding author upon request.

## References

[1] Mandel J.J., Yust-Katz S., Wu J., Yuan Y., Webre C.C., Pawar H.L.T., Gilbert M.R., Armstrong T.S. (2014). Venous thromboembolism (VTE) and glioblastoma. J. Clin. Oncol..

[2] Edwin N.C., Elson P., Ahluwalia M.S., Khorana A.A. (2015). Venous thromboembolism in patients with glioblastoma, risk factors and prognostic importance. J. Clin. Oncol..

[3] Takano S., Tsuboi K., Tomono Y., Mitsui Y., Nose T. (2000). Tissue factor, osteopontin, αυβ3 integrin expression in microvasculature of gliomas associated with vascular endothelial growth factor expression. Br. J. Cancer.

[4] Fadul C.E., Zacharski L.R. (2005). Coagulation biology in glioma pathogenesis, a missing link?. J. Throm. Haemost..

[5] Wojtukiewicz M.Z., Sierko E., Rak J. (2004). Contribution of the hemostatic system to angiogenesis in cancer. Semin. Thromb. Hemost..

[6] Ruf W., Muller B.M. (1996). Tissue factor in cancer angiogenesis and metastasis. Curr. Opin. Hematol..

[7] Ornstein D.L., Zacharski L. (2002). The coagulation system as a target for the treatment of human gliomas. Semin. Thromb. Hemost..

[8] Bardos H., Molnar P., Csecsei G., Adany R. (1996). Fibrin deposition in primary and metastatic human brain tumors. Blood Coagul. Fibrinolysis..

[9] Broze G.J. (1995). Tissue factor pathway inhibitor. Thromb. Haemost..

[10] Gerlach R., Scheuer T., Bohm M., Beck J., Woszczyk A., Raabe A., Scharrer I., Seifert V. (2003). Increased levels of plasma tissue factor pathway inhibitor in patients with glioblastoma and intracerebral metastases. Neurol. Res..

[11] Radziwon P., Schenk J.F., Mazgajska K., Boczkowska-Radziwon B., Galar M., Kloczko J., Wojtukiewicz M.Z. (2002). Tissue factor (TF) and inhibitor (TFPI) concentrations in patients with urinary tract tumors and haematological malignancies. [in Polish]. Pol. Merkur. Lekarski..

[12] Sierko E., Wojtukiewicz M.Z., Zimnoch L., Kisiel W. (2010). Expression of tissue factor pathway inhibitor (TFPI) in human breast and colon cancer tissue. Thromb. Haemost..

[13] Hamamoto T., Yamamoto M., Nordfang O., Petersen J.G., Foster D.C., Kisiel W. (1993). Inhibitory properties of full-length and truncated recombinant tissue factor pathway inhibitor (TFPI). J. Biol. Chem..

[14] Iversen L.H., Okholm M., Thorlacius-Ussing O.O. (1996). Pre- and postoperative state of coagulation and fibrinolysis in plasma of patients with benign and malignant colorectal disease—A preliminary study. Thromb. Haemost..

[15] Erman M., Abali H., Oran B., Haznedaroglu I.C., Canpinar H., Kirazli S., Celik I. (2004). Tamoxifen-induced tissue factor pathway inhibitor reduction, a clue for an acquired thrombophilic state?. Ann. Oncol..

[16] Udagawa K., Miyagi Y., Hirahara F., Miyagi E., Nagashima Y., Minaguchi H., Misugi K., Yasumitsu H., Miyazaki K. (1998). Specific expression of PP5/TFPI-2 mRNA by syncytiotrophoblasts in human placenta as revealed by in situ hybridization. Placenta.

[17] Izumi H., Takahashi C., Oh J., Noda M. (2000). Tissue factor pathway inhibitor-2 suppresses the production of active matrix metalloproteinase-2 and is down-regulated in cells harboring activated ras oncogene. FEBS Lett..

[18] Saito E., Okamoto A., Saito M., Shinozaki H., Takakura S., Yanaihara N., Ochiai K., Tanaka T. (2005). Genes associated with the genesis of leiomyoma of the uterus in a commonly deleted chromosomal region at 7q22. Oncol. Rep..

[19] George J., Gondi C.S., Dinh D.H., Gujrati M., Rao J.S. (2007). Restoration of tissue factor patway inhibitor-2 in a human glioblastoma cell line triggers caspase-mediated pathway and apoptosis. Clin. Cancer Res..

[20] Yanamandra N., Kondraganti S., Gondi C.S., Gujrati M., Olivero W.C., Dinh D.H., Rao J.S. (2005). Recombinant adeno-associated virus (rAAV) expressing TFPI-2 inhibits invasion, angiogenesis and tumor growth in a human glioblastoma cell line. Int. J. Cancer.

[21] Ivanciu L., Gerard R.D., Tang H., Lupu F., Lupu C. (2007). Adenovirus-mediated expression of tissue factor pathway inhibitor-2 inhibits endothelial cell migration and angiogenesis. Arterioscler. Thromb. Vasc. Biol..

[22] Esmon C.T. (2000). The endothelial cell protein C receptor. Thromb. Haemost..

[23] Esmon C.T. (2001). Role of coagulation inhibitors in inflammation. Thromb. Haemost..

[24] Furuta J., Kaneda A., Umebayashi Y., Otsuka F., Sugimura T., Ushijama T. (2005). Silencing of the thrombomodulin gene in human malignant melanoma. Melanoma Res..

[25] Zhang Y., Weiler-Guettler H., Chen J., Wilhelm O., Deng Y., Qiu F., Nakagawa K., Klevesath M., Wilhelm S., Böhrer H. (1998). Thrombomodulin modulates growth of tumor cells independent of its anticoagulant activity. J. Clin. Investig..

[26] Benzakour O., Kanthou C. (2000). The anticoagulant factor, protein S, is produced by cultured human vascular smooth muscle cells and its expression is up-regulated by thrombin. Blood.

[27] Kanthou C., Benzakour O. (2000). Cellular effects and signalling pathways activated by the anti-coagulant factor, protein S, in vascular cells protein S cellular effects. Adv. Exp. Med. Biol..

[28] Lindahl A.K., Sandset P.M., Abildgaard U., Adersson T.R., Harbitz T.B. (1989). High plasma levels of extrinsic pathway inhibitor and low levels of other coagulation inhibitors in advanced cancer. Acta Chir. Scand..

[29] Pavon M.A., Arroyo-Solera I., Téllez-Gabriel M., Leon X., Viros D., Lopez M., Gallardo A., Cespedes M.V., Casanova I., López-Pousa A. (2015). Enhanced cell migration and apoptosis resistance may underlie the association between high SERPINE1 expression and poor outcome in head and neck carcinoma patients. Oncotarget.

[30] Sakakibara T., Hibi K., Koike M., Fujiwara M., Kodera Y., Ito K., Nakao A. (2005). Plasminogen activator inhibitor-1 as a potential marker for the malignancy of colorectal cancer. Br. J. Cancer.

[31] Becker M., Szarvas T., Wittschier M., vom Dorp F., Tötsch M., Schmid K.W., Rübben H., Ergün S. (2010). Prognostic impact of plasminogen activator inhibitor type 1 expression in bladder cancer. Cancer.

[32] Xi X., Liu N., Wang Q., Chu Y., Yin Z., Ding Y., Lu Y. (2019). ACT001, a novel PAI-1 inhibitor; exerts synergistic effects in combination with cisplatin by inhibiting PI3K/AKT pathway in glioma. Cell Death Dis..

[33] Blasi F. (1999). Proteolysis, cell adhesion, chemotaxis and invasiveness are regulated by the u-PA-u-PAR-PAI-1 system. Thromb. Haemost..

[34] Li S., Wei X., He J., Tian X., Yuan S., Sun L. (2018). Plasminogen activator inhibitor-1 in cancer research. Biomed. Pharmacother..

[35] Placencio V.R., DeClerck Y.A. (2015). Plasminogen activator inhibitor-1 in cancer: Rationale and insight for future therapeutic testing. Cancer Res..

[36] Pavon M.A., Arroyo-Solera I., Cespedes M.V., Casanova I., Leon X., Mangues R. (2016). uPA/uPAR and SERPINE1 in head and neck cancer: Role in tumor resistance, metastasis, prognosis and therapy. Oncotarget.

[37] Fang H., Jin J., Huang D., Yang F., Guan X. (2018). PAI-1 induces Src inhibitor resistance via CCL5 in HER2-positive breast cancer cells. Cancer Sci..

[38] Kit O.I., Frantsiyants E.M., Kozlova L.S., Rostorguev E.E., Balyazin-Parfenov V., Pogorelova Y.A. (2017). A plasminogen regulation system in brain tumors. Zhurnal Vopr. Neirokhirurgii Imeni N. N. Burdenko.

[39] De Candida E. (2012). Mechanisms of platelet activation by thrombin, a short history. Thromb. Res..

[40] Ay C., Vormittag R., Dunkler D., Simanek R., Chiriac A.L., Drach J., Quehenberger P., Wagner O., Zielinski C., Pabinger I. (2009). D-dimer and prothrombin fragment 1 + 2 predict venous thromboembolism in patients with cancer, results from the Vienna Cancer and Thrombosis Study. J. Clin. Oncol..

[41] Wojtukiewicz M.Z., Rucinska M., Zimnoch L., Jaromin J., Piotrowski Z., Rozanska-Kudelska M., Kisiel W., Kudryk B.J. (2000). Expression of prothrombin fragment 1+2 in cancer tissue as an indicator of local activation of blood coagulation. Thromb. Res..

[42] Ota S., Wada H., Abe Y., Yamada E., Sakaguchi A., Nishioka J., Hatada T., Ishikura K., Yamada N., Sudo A. (2008). Elevated levels of prothrombin fragment 1 + 2 indicate high risk of thrombosis. Clin. Appl. Thromb. Hemost..

[43] Hsu S., Raine L., Fanger H. (1981). Use of avidin-biotin-peroxidase complex (ABC) in immunoperoxidase techniques, a comparison between ABC and unlabeled antibody (PAP) procedures. J. Histochem. Cytochem..

[44] Sierko E., Wojtukiewicz M.Z., Zawadzki R., Zimnoch L., Kisiel W. (2010). Expression of protein C (PC), protein S (PS), and thrombomodulin (TM) in loco in human colorectal cancer. Thromb. Res..

[45] Wojtukiewicz M.Z., Sierko E., Zimnoch L., Kozłowski L., Sulkowski S., Kisiel W. (2003). Immunohistochemical localization of tissue factor pathway inhibitor-2 in human tumor tissue. Thromb. Haemost..

[46] Hirsch F.R., Varella-Garcia M., Bunn P.A., Di Maria M.V., Veve R., Bremmes R.M., Barón A.E., Zeng C., Franklin W.A. (2003). Epidermal growth factor receptor in non-small-cell lung carcinomas, correlation between gene copy number and protein expression and impact on prognosis. J. Clin. Oncol..

[47] Pirker R., Pereira J.R., von Pawel J., Krzakowski M., Ramlau R., Park K., de Marinis F., Eberhardt W.E., Paz-Ares L., Störkel S. (2012). EGFR expression as a predictor of survival for first-line chemotherapy plus cetuximab in patients with advanced non-small-cell lung cancer, analysis of data from the phase 3 FLEX study. Lancet Oncol..

[48] Ota Y., Koizume S., Nakamura Y., Yoshihara M., Takahashi T., Sato S., Myoba S., Ohtake N., Kato H., Yokose T. (2021). Tissue factor pathway inhibitor-2 is specifically expressed in ovarian clear cell carcinoma tissues in the nucleus, cytoplasm and extracellular matrix. Ocol. Rep..

[49] Shimizu K., Matsumoto H., Hirata H., Ueno K., Samoto M., Mori J., Fujii N., Kawai Y., Inoue R., Yamamoto Y. (2020). ARHGAP29 expression may be a novel prognostic factor of cell proliferation and invasion in prostate cancer. Ocol. Rep..

[50] Wojtukiewicz M.Z., Hempel D., Sierko E., Tucker S.C., Honn K.V. (2016). Thrombin—unique coagulation system protein with multifaceted impacts on cancer and metastasis. Cancer Met. Rev..

[51] Tawil N., Bassawon R., Rak J. (2019). Oncogenes and clotting factors, the emerging role of tumor cell genome and epigenome in cancer-associated thrombosis. Semin. Thromb. Hemost..

[52] Tawil N., Spinelli C., Bassawon R., Rak J. (2020). Genetic and epigenetic regulation of cancer coagulome—Lessons from heterogeneity of cancer cell populations. Thromb. Res..

[53] Wojtukiewicz M.Z., Sierko E., Kisiel W. (2007). The role of hemostatic system inhibitors in malignancy. Semin. Thromb. Hemost..

[54] Sierko E., Wojtukiewicz M.Z., Zimnoch L., Thorpe P.E., Brekken R.A., Kisiel W. (2011). Co-localization of prothrombin fragment F1+2 and VEGF-R2-bound VEGF in human colon cancer. Anticancer Res..

[55] Falanga A., Schieppati F., Russo D. (2015). Cancer tissue procoagulant mechanisms and the hypercoagulable state of patients with cancer. Semin. Thromb. Hemost..

[56] Pindon A., Hantai D., Jandrot-Perrus M., Festoff B.W. (1997). Novel expression and localization of active thrombomodulin on the surface of mouse brain astrocytes. Glia.

[57] Tsuneyoshi N., Fukudome K., Horiguchi S., Ye X., Matsuzaki M., Toi M., Suzuki K., Kimoto M. (2001). Expression and anticoagulant function of the endothelial cell protein C receptor (EPCR) in cancer cell lines. Thromb. Haemost..

[58] Shua F., Kobayashia H., Fukudomeb K., Tsuneyoshib N., Kimotob M. (2000). Activated protein C suppresses tissue factor expression on U937 cells in the endothelial protein C receptor-dependent manner. FEBS Lett..

[59] Suzuki K., Hayashi T. (2007). Protein C and its inhibitor in malignancy. Semin. Thromb. Hemost..

[60] Philips D.J., Greengard J.S., Fernandez J.A., Ribeiro M., Evatt B.L., Griffin J.H., Hooper W.C. (1993). Protein S, an antithrombotic factor, is synthesized and released by neural tumor cells. J. Neurochem..

[61] Wimmel A., Rohner I., Ramaswamy A., Heidtmann H.H., Seitz R., Kraus M., Schuermann M. (1999). Synthesis and secretion of the anticoagulant protein S and coexpression of the Tyro3 receptor in human lung carcinoma cells. Cancer.

[62] Wojtukiewicz M.Z., Zacharski L.R., Memoli V.A., Kisiel W., Kudryk B.J., Rousseau S.M.D., Stump C. (1990). Malignant melanoma. Interaction with coagulation and fibrinolysis pathways in situ. Am. J. Clin. Pathol..

[63] Wojtukiewicz M.Z., Zacharski L.R., Memoli V.A., Kisiel W., Kudryk B.J., Rousseau S.M., Moritz T.E., Stump D.C. (1991). Fibrin formation on vessel walls in hyperplastic and malignant prostate tissue. Cancer.

[64] Wojtukiewicz M.Z., Sierko E., Zacharski L.R., Zimnoch L., Kudryk B., Kisiel B. (2003). Tissue factor-dependent coagulation activation and impaired fibrinolysis in situ in gastric cancer. Semin. Thromb. Hemost..

[65] Wojtukiewicz M.Z., Rucińska M., Zacharski L.R., Kozlowski L., Zimnoch L., Piotrowski Z., Kudryk B.J., Kisiel W. (2001). Localization of blood coagulation factors in situ in pancreatic carcinoma. Thromb. Haemost..

[66] Wizigmann-Voos S., Plate K.H. (1996). Pathology, genetics and cell biology of hemangioblastomas. Histol. Histopathol..

[67] Kafri N.A., Hafizi S. (2019). Tumour-secreted protein S (ProS1) activates a Tyro3-Erk signalling axis and protects cancer cells from apoptosis. Cancers.

[68] Lai C., Gore M., Lemke G. (1994). Structure, expression, and activity of Tyro-3, a neural adhesion-related receptor tyrosine kinase. Oncogene.

[69] Werling R.W., Zacharski L.R., Kisiel W., Bajaj S.P., Memoli V.A., Rousseau S.A. (1993). Distribution of tissue factor pathway inhibitor in normal and malignant human tissues. Thromb. Haemost..

[70] Kurer M.A. (2007). Protein and mRNA expression of tissue factor pathaway inhibitor-1 (TFPI-1) in breast, pancreatic and colorectal cancer cells. Mol. Biol. Rep..

[71] Kageshita T., Funasaka Y., Ichihashi M., Ishichara T., Tokuo H., Ono T. (2002). Differential expression of tissue factor and tissue factor pathway inhibitor in metastatic melanoma lesions. Pigment Cell Res..

[72] Ruf W., Seftor E.A., Petrovan R.J., Weiss R.M., Gruman L.M., Margaryan N.V., Seftor R.E.B., Miyagi J., Hendrix M.J.C. (2003). Differential role of tissue factor pathway. Cancer Res..

[73] Wojtukiewicz M.Z., Mysliwiec M., Matuszewska E., Sulkowski S., Zimnoch L., Politynska B., Wojtukiewicz A.M., Tucker S.C., Honn K.V. (2021). Heterogeneous expression of proangiogenic and coagulation proteins in gliomas of different histopathological grade. Pathol Onco Res..

[74] Takeshima H., Nishi T., Kuratsu J., Kamikubo Y., Kochi M., Ushio Y. (2000). Suppression of the tissue factor-dependent coagulation cascade: A contributing factor for the development of intratumoral hemorrhage in glioblastoma. Int. J. Mol. Med..

[75] Amirkhoshravi A., Meyer T., Chang J.-Y., Amaya M., Siddiqui H., Francis J.L. (2002). Tissue factor pathway inhibitor reduces experimental lung metastases of B16 melanoma. Thromb. Haemost..

[76] Rao C.N., Lakka S.S., Kin Y., Konduri S.D., Fuller G.N., Mohanam S., Rao J.S. (2001). Expression of tissue factor pathway inhibitor 2 inversely correlates during the progression of human gliomas. Clin. Cancer Res..

[77] Sato N., Parker A.R., Fukushima N., Miyagi Y., Iacobuzio-Donahue C.A., Eshleman J.R., Goggins M. (2005). Epigenetic inactivation of TFPI-2 as a mechanism associated with growth and invasion of pancreatic ductal adenocarcinoma. Oncogene.

[78] Wang G., Huang W., Li W., Chen S., Chen W., Zhou Y., Peng P., Gu W. (2018). TFPI-2 suppresses breast cancer cell proliferation and invasion through regulation of ERK signaling and interaction with actinin-4 and myosin-9. Sci. Rep..

[79] Dong J.T. (2001). Chromosomal deletions and tumor suppressor genes in prostate cancer. Cancer Metast. Rev..

[80] Sell S.M., Tullis C., Stracner D., Song C.Y., Gewin J. (2005). Minimal interval defined on 7q in uterine leiomyoma. Cancer Genet. Cytogenet..

[81] IzCaccamo D.V., Keohane M.E., McKeever P.E. (1994). Plasminogen activators and inhibitors in gliomas, an immunohistochemical study. Mod. Pathol..

[82] Kono S., Rao J.S., Bruner J.M., Sawaya R. (1994). Immunohistochemical localization of plazminogen activator inhibitor type 1 in human brain tumors. J. Neuropathol. Exp. Neurol..

[83] Yamamoto M., Sawaya R., Mohanam S., Loskutoff D.J., Bruner J.M., Rao V.H., Oka K., Tomonga M., Nicolson G.L., Rao J.S. (1994). Expression of cellular localization of messenger RNA for plasminogen activator inhibitor type-1 in human astorcytoma in vivo. Cancer Res..

[84] Muracciole X., Romain S., Dufour H., Palmari J., Chinot O., Ouafik L., Grisoli F., Branger D.F., Martin P.M. (2002). PAI-1 and EGFR expression in adult glioma tumors, toward a mole prognostic classification. Int. J. Radiat. Oncol. Biol. Phys..

[85] Sawaya R., Yamamoto M., Rama O.J., Shi M.L., Rayford A., Rao J.S. (1995). Plasminogen activator inhibitor-1 in brain tumors, relation to malignancy and necrosis. Neurosurgery.

